# Diagnosis of GH Deficiency Without GH Stimulation Tests

**DOI:** 10.3389/fendo.2022.853290

**Published:** 2022-02-18

**Authors:** Anastasia Ibba, Sandro Loche

**Affiliations:** Struttura Semplice Dipartimentale (SSD) Endocrinologia Pediatrica e Centro Screening Neonatale, Ospedale Pediatrico Microcitemico “A. Cao”, Azienda di Rilievo Nazionale ed Alta Specializzazione (ARNAS) G. Brotzu, Cagliari, Italy

**Keywords:** children, growth, growth hormone, IGF-1, growth hormone stimulation tests, newborn

## Abstract

Growth hormone deficiency (GHD) is the most commonly affected pituitary hormone in childhood with a prevalence of 1 in 4000–10000 live births. GH stimulation testing (GHST) is commonly used in the diagnostic workup of GHD. However, GHD can be diagnosed in some clinical conditions without the need of GHST. The diagnosis of GHD in newborns does not require stimulation testing. Likewise infants/children with delayed growth and/or short stature associated with neuroradiological abnormalities and one or more additional pituitary hormone deficiencies may not need GHST. This review summarizes the current evidence on the diagnosis of GHD without stimulation tests.

## Introduction

Growth hormone deficiency (GHD) has a prevalence of 1:4000-10000, most cases are isolated (IGHD) and the majority of them are idiopathic. GHD can also be combined with other pituitary hormone deficiencies (multiple pituitary hormone deficiency-MPHD). Both IGHD and MPHD can be congenital or acquired (tumours, trauma, brain infections, radiotherapy). Congenital IGHD can be due to genetic mutations in the genes encoding GH (GH1) or the GH-releasing hormone receptor (GHRHR) (1, 2). Mutations in the genes encoding transcription factors like SOX3, HESX1, GLI2, OTX2, LHX3, LHX4, PROP1, and POU1F1 usually cause MPHD (3) but, occasionally, GHD can be the only pituitary hormone deficiency (1, 2). GHD can develop at any age, so the signs and symptoms vary accordingly. GHD in newborns may present with hypoglycaemia, jaundice, or with underdeveloped male genitalia, whereas in older children it manifests primarily with short stature and/or decreased growth (4).

In the last decades the diagnosis of idiopathic IGHD has been the subject of intensive debate and it is still controversial. GH stimulation testing is not necessary in neonates and also in infants with a combination of clinical signs of GHD, low insulin-like growth factor 1 (IGF-1) and IGF binding protein-3 (IGFBP-3), MPHD and/or an abnormal brain magnetic resonance imaging (MRI) (5, 6).

The most recent guidelines (7) still recommend to perform GHSTs in children and adolescents with suspected GHD. However, some authors (8, 9) suggest that also in this age group the diagnosis of GHD may be based on a combination of auxological, biochemical (IGF-1 and IGFBP-3), neuroradiological and genetic findings and that GHSTs are not always necessary. It should be pointed out that the diagnosis of GHD is primarily auxologic (10, 11).

In this review we summarize the current evidence on the diagnosis of GHD without the use of GHSTs in the paediatric population.

## Physiopathology

GH is a 191-amino acid protein synthesized, stored and secreted in a pulsatile manner by pituitary somatotroph cells. The synthesis and release of GH are under the control of various hormones, including GH-releasing hormone (GHRH), somatostatin, ghrelin, IGF-1, thyroid hormone, gonadal steroids and glucocorticoids. At birth and in the first week of life, GH levels are high and pulsatile, with elevated baseline, mean and peak levels (4) and rapidly decrease during the following weeks and increase with chronic malnutrition, chronic kidney disease, exercise, trauma and sepsis (12). GH plays a key role in glucose and fat metabolism in the newborn (4, 13), in increasing bone length and density in children and adolescents, but also in increasing muscle mass, regulating lipid and carbohydrates metabolism and body water throughout life. GH action is exerted directly on target tissues or indirectly by insulin growth factors (IGFs) (14). IGF-1, the main GH effector, is mostly secreted by the liver, and circulates bound to specific insulin growth factor binding protein (IGFBPs 1-6), mainly IGFBP-3. IGF-1 secretion is influenced also by malnutrition, thyroid hormone, sex hormones, chronic diseases and inflammation and anorexia nervosa (15–19). In contrast, IGF-1 values remain low for at least the first 15–18 months of age and increase until a pubertal peak (20, 21). Measurement of a random serum GH level is not helpful for the diagnosis of GHD except in some specific cases (see below). In fact, serum GH levels between normal pulses of GH secretion, are often low, below the limits of sensitivity of most conventional assays. For these reasons stimulation tests have been introduced in the diagnostic workup of GHD many years ago. A large number of GHSTs have been proposed in the last decades (6, 22–25). However, GHSTs are not physiological, have poor specificity and reproducibility (24), and cause a high number of false pathological responses. Furthermore, there are no age- and gender-related normative data, and the diagnostic cut-offs are often arbitrarily established. In addition, GH secretion is influenced by several factors (such obesity, undernutrition, puberty) and entails high costs and discomfort for the patients. Notwithstanding the above limitations, GHSTs are still used as the gold standard for the diagnosis of paediatric GHD (7, 26–29). These limitations are even more evident in children younger than 4 years, in which the accuracy of stimulation tests has not been formally addressed, and most of the currently used tests are burdened by side effects (5, 24). For all the above reasons the decision to perform a GHST should be well reasoned and based on the severity of short stature, height velocity, medical history and physical examination findings. The diagnostic GH peak cut-off is still matter of discussion between scientific societies, and so far it has been arbitrarily established by the single centre and currently ranges between 3 to 10 μg/L (6, 7, 11, 22, 24, 27, 30–32).

## Clinical Presentation of GHD

### Newborn

In consideration of the important metabolic role of GH in the neonatal period, the prompt identification of a newborn with GHD is crucial to start replacement treatment rapidly. GHD in neonates can be isolated but often presents as MPHD, and the clinical presentation and its severity depend on the number of affected hormones. Neonates might present non-specific symptoms and signs, such as lethargy and poor weight gain, or more specific life-threatening emergencies (33), including respiratory distress, apnoea, cyanosis, poor feeding, hypotonia, long-term cholestatic jaundice, severe hypoglycaemia with or without seizures, temperature dysregulation, electrolyte abnormalities, haemodynamic instability and/or neonatal sepsis. Other physical findings can suggest the presence of GHD such as eye abnormalities, microphallus, microphthalmia and single central maxillary incisor. Intrauterine growth is generally not affected by GHD, and birth weight and length are usually within normal limits, although slightly reduced.

### Infant/Child

Short stature, defined as a height more than 2 SD below the population mean, or growth arrest/deceleration with normal/increased weight and delayed skeletal maturation may be the only signs of GHD in infancy and childhood. Diminished height velocity often precedes short stature. The typical GHD clinical phenotype in infants is persistent growth failure and/or short stature associated with truncal adiposity and micropenis, immature appearance, mid-facial hypoplasia, delayed dentition and frontal bossing with depressed nasal bridge.

Most cases of IGHD in childhood are idiopathic. However, pituitary masses, brain tumours, infections of central nervous system should always be ruled out. Furthermore, GHD should be suspected in short children who underwent cranial irradiation (34, 35) or suffered from brain injuries (36).

## Establishing a Diagnosis

### Newborn

The neonatal period is characterized by high GH levels (hypersomatotropism of the newborn) (37, 38), which enable the diagnosis of GHD without the use of pharmacological stimulation (20, 39). Furthermore GHSTs are contraindicated under the age of 1-2 years (2). Reasons for this are primarily due to safety (GHSTs need a fasting period), to the amount of time needed, to the potential for hypoglycaemia, or other side effects depending on the GH secretagogue used.

A single low GH measurement is traditionally used to confirm the clinical suspicion of neonatal GHD. The sample is preferably taken during hypoglycaemic episodes (critical sample), in plasma, serum or newborn screening cards (39) within the first week of life. However, the specificity of a single GH measurement during spontaneous hypoglycaemia has been questioned, and some authors (40) do not deem it sufficient to diagnose GHD. However, the observation of normal GH concentration can be useful to exclude GHD. Over the years, various cut-offs of GH as indicative of GH sufficiency in the newborn have been proposed ranging from 7 to 20 µg/L during an hypoglycaemic episode (20, 22). Recently, Binder et al. (39) demonstrated that GH concentration <7 μg/L in the newborn screening card confirms severe GHD with high accuracy in term newborns with a specific phenotype such as recurrent hypoglycaemia, additional pituitary hormone deficiencies and/or a significant hypothalamic-pituitary abnormality on cerebral MRI (**Table 1**). The use of newborn screening card still needs to be validated since its reliability has not been confirmed (41). According to current guidelines (7) the diagnosis of GHD in newborns is possible in the presence of GH concentrations ≤5 ng/mL in a newborn with additional pituitary hormone deficiencies or/and the triad of ectopic posterior pituitary, pituitary hypoplasia and abnormal pituitary stalk (**Table 1**).

**Table 1:** Conditions in which it is not necessary to perform GHSTs according to current guidelines (6, 7, 22, 39).

**Newborns**	GH ≤ 7 μg/L during hypoglycaemia episode with a specific phenotype
	Random GH ≤ 5 ng/mL with additional pituitary hormone deficiencies or/and the triad of ectopic posterior pituitary, pituitary hypoplasia and abnormal stalk
**Infants**	Suggestive history of clinical GHD (short stature and/or low height velocity), low IGF-1 and IGFBP-3, multiple pituitary hormone deficiencies, and/or an abnormal cranial MRI
**Children**	Auxological characteristics (short stature and/or low height velocity), presence of hypothalamic-pituitary defects (congenital or acquired), and one additional pituitary hormone deficiency

### Infant/Child

An accurate and early diagnosis is important for a prompt treatment initiation, essential to optimize child growth and adult height and to avoid co-morbidities such as impaired quality of life, bone and metabolic health (7, 26). Despite more than 50 years of paediatric hGH replacement, the ability to make a definitive diagnosis of GHD in children is still limited. The diagnosis of GHD is traditionally based on auxology and the lack of response to two different GHSTs, but it is not always straightforward (10).

In infants with history of hypoglycaemia, hyperbilirubinemia, poor growth, midline defects, microphallus, low IGF-1 and IGFBP-3, MPHD, such as TSH and ACTH deficiency, and/or an abnormal brain MRI, the diagnosis of GHD is possible without stimulation test (6) (**Table 1**).

According to recent guidelines (7) a diagnosis of GHD without GHSTs in children is suggested only in subjects that fulfil all the following criteria: auxological characteristics, presence of hypothalamic-pituitary defects (congenital or acquired), and one additional pituitary hormone deficiency (**Table 1**). However, according to some authors (26) there are more conditions in which GHSTs might be not necessary, such as in case of acquired GHD due to intracranial tumours, severe traumatic brain injury or cranial radiotherapy (**Table 2**). Given to the lack of sufficient evidence, the guidelines do not recommend establishing the diagnosis of GHD without GHSTs in patients with these conditions (7, 34, 35). Due to the low reliability of the GHSTs, alternative diagnostic strategies such as measurement of IGF-1 and IGFBP-3, genetic testing and neuroimaging have been considered over the years for the diagnosis of GHD in children (5, 8, 9, 42). In our opinion, patients with auxological characteristics associated with abnormal hypothalamic/pituitary morphology and low IGF-1 do not require GHST.

**Table 2:** Conditions in which it is not necessary to perform GHSTs. Modified from Clément et al. (26).

Auxological criteria required for performing GHSTs according to the Summary Statement of the Growth Hormone Research Society (22):	1) severe short stature, defined as a height more than 3 SD below the mean; 2) height more than 1.5 SD below the midparental height; 3) height more than 2 SD below the mean and a height velocity over 1 year more than 1 SD below the mean for chronological age, or a decrease in height SD of more than 0.5 over 1 year in children over 2 year of age; 4) in the absence of short stature, a height velocity more than 2 SD below the mean over 1 year or more than 1.5 SD sustained over 2 year; 5) signs indicative of an intracranial lesion; 6) signs of MPHD; 7) neonatal symptoms and signs of GHD.
**PLUS**	
Pituitary dysgenesis on MRI or Two or more anterior pituitary hormone deficiencies or At least one anterior pituitary hormone deficiency plus one of the following: a. Neonatal symptoms of pituitary deficiency (hypoglycaemia or hypogenitalism) b. Central diabetes insipidus c. Clinical or radiological craniofacial midline abnormalities d. Suprasellar or sellar tumor/surgery e. Cranial radiotherapy ≥18 Gy	

Recently Clément et al. (26) developed and validated an accurate clinical prediction rule for the diagnosis of GHD without GHSTs in children who meet the criteria required for GHSTs according to the guidelines (22), but with specific comorbidities such as the presence of pituitary dysgenesis on MRI or two or more anterior pituitary hormone deficiencies (**Table 2**).

However, recent guidelines (6) still consider the measurements of IGF-1, IGFBP-3 levels, brain MRI and genetic tests only as a support for the diagnosis.

#### IGF-1 and IGFBP-3

Measurement of IGF-1 is considered not useful in newborns since its levels remain low for at least the first 15–18 months of age and then progressively increase reaching a peak at mid-puberty (20, 21). The usefulness of IGF-1 measurement in children, alone or in combination with IGFBP-3, for the diagnosis of GHD has been the subject of a number of studies (7, 18, 23, 28, 30, 31, 42–50). The results of all these studies have been controversial, and their findings are hardly comparable because of the use of different assays, different measure unit (μg/ml, percentiles, SDS), as well as patients’ selection (43). However, most studies showed poor sensitivity and specificity in the diagnosis of GHD in children and most authors concluded that IGF-1 measurement is useful for the diagnosis of GHD only when combined with auxological parameters and the results of GHSTs (31, 42, 44, 46, 51–53). IGF-1 levels should be interpreted taking into consideration age, gender, pubertal status and body mass index (18). Moreover reduced IGF-1 levels may be observed children with malnutrition (19), hypothyroidism, hepatic disease or diabetes mellitus and there is overlap between normal and GHD children. Therefore, although very low levels of IGF-1 are strongly suggestive of GHD, normal IGF-1 concentrations do not exclude GHD at any age (54). Wit et al. (18) recently proposed specific steps for the clinicians for the use of IGF-1 measurement to estimate the probability of GHD in a child with growth failure based on *pre-* and *post-test likelihood.* In our personal experience about 40% of patients with severe GHD have IGF-1 concentrations higher than -2 SDS, overlapping values found in non-GHD children (53).

IGFBP-3 levels have also been considered for the diagnosis of GHD since it is less influenced by nutrition than IGF-1. However, many studies have reported no advantage of measuring IGFBP-3 over IGF-1 (55).

#### Magnetic Resonance Imaging

The differential diagnosis of hypopituitarism has greatly improved thanks to diagnostic accuracy of MRI that has increased our knowledge of pituitary morphology and function (56, 57). Brain MRI with a focus on the pituitary and hypothalamus is essential during the initial evaluation of newborns with midline defects, microphallus, and hypoglycaemia. In a infant with a highly suspicious history of GHD, plus other pituitary hormone deficiencies, or neurologic abnormalities, the presence of an abnormal brain MRI allows the diagnosis of GHD without GHSTs (6). Abnormalities found on MRI that are more suggestive of GHD include the absence of the anterior pituitary gland (empty sella), an ectopic posterior pituitary gland, and hypoplasia/absence of the pituitary stalk and/or pituitary gland (58). However the presence of a small pituitary gland by itself is not sufficient to make the diagnosis of GHD, but it may suggest the need for a more extensive evaluation of pituitary function (6). In children younger than 4 years, MRI has been proposed as first-line investigation (5) in order to reduce cost/benefit ratio and allow earlier start of treatment, and to postpone GHSTs to an age when they can be more easily performed and interpreted. Neuroimaging in association with IGF-1 assessment has been proposed about 20 years ago in children with clinical suspicion of GHD as an alternative to GHSTs (8). However the current guidelines (6) still recommend to perform MRI of the hypothalamus and pituitary after the diagnosis of GHD is confirmed by GHSTs. Therefore, if GHD has been excluded by GHSTs, MRI is typically not indicated.

#### Genetic Testing

Genetic and/or epigenetic testing is not required for all suspects of GHD but it is suggested in the diagnostic assessment of a patient whose phenotype suggests a high likelihood of a genetic cause (6) such as in case of suspected congenital hypopituitarism, early onset of growth failure, positive family history, height more than 3 SD below the mean, extremely low GH response to GHSTs and, very low IGF-1 and IGFBP-3 levels. The most common mutations in patients with isolated GHD have been identified in GH1 and GHRHR genes and may be associated with a normal MRI scan. Other gene mutations (i.e. POU1F1, PROP1, LHX3, LHX4, HESX1, SOX2, SOX3, etc.) are generally associated with MPHD (2), and present with typical clinical and neuroradiological features. With a greatly use of genetic testing it is possible that other conditions may include GHD in the differential diagnosis (59).

## Conclusions

In most cases of suspected GHD current guidelines still recommend the use of GHSTs plus auxological criteria. However, GHSTs are not accurate, and in some instances a diagnosis can be made based on other clinical, laboratory, genetic and neuroimaging evaluation. IGF-1 and IGFBP-3 measurement have high specificity but low sensitivity and thus normal concentrations do not exclude GHD at any age. MRI of the hypothalamic–pituitary region might be helpful in identifying GHD when associated with other cerebral abnormalities, and genetic testing can provide definitive diagnosis in some selected patients. The diagnosis of GHD may be straightforward in neonates, infants and children with organic lesions, irradiation or trauma, but is still puzzling in all other conditions, requiring careful clinical, laboratory and imaging investigation.

## Author Contributions

Both authors contributed equally to the design and writing of the review. All authors contributed to the article and approved the submitted version.

## Conflict of Interest

The authors declare that the research was conducted in the absence of any commercial or financial relationships that could be construed as a potential conflict of interest.

## Publisher’s Note

All claims expressed in this article are solely those of the authors and do not necessarily represent those of their affiliated organizations, or those of the publisher, the editors and the reviewers. Any product that may be evaluated in this article, or claim that may be made by its manufacturer, is not guaranteed or endorsed by the publisher.

## References

[1] Alatzoglou KS Webb EA Le Tissier P Dattani MT. Isolated Growth Hormone Deficiency (GHD) in Childhood and Adolescence: Recent Advances. Endocrine Rev (2014) 35:376–432. doi: 10.1210/er.2013-1067 24450934

[2] Bosch i Ara L Katugampola H Dattani MT. Congenital Hypopituitarism During the Neonatal Period: Epidemiology, Pathogenesis, Therapeutic Options, and Outcome. Front Pediatr (2021) 8:600962. doi: 10.3389/fped.2020.600962 33634051 PMC7902025

[3] Alatzoglou KS Dattani MT. Genetic Forms of Hypopituitarism and Their Manifestation in the Neonatal Period. Early Hum Dev (2009) 85:705–12. doi: 10.1016/j.earlhumdev.2009.08.057 19762173

[4] Ogilvy-Stuart AL. Growth Hormone Deficiency (GHD) From Birth to 2 Years of Age: Diagnostic Specifics of GHD During the Early Phase of Life. Horm Res Paediatr (2003) 60:2–9. doi: 10.1159/000071219 12955011

[5] Pampanini V Pedicelli S Gubinelli J Scirè G Cappa M Boscherini B. Brain Magnetic Resonance Imaging as First-Line Investigation for Growth Hormone Deficiency Diagnosis in Early Childhood. Horm Res Paediatr (2015) 84:323–30. doi: 10.1159/000439590 26393500

[6] Collett-Solberg PF Ambler G Backeljauw PF Bidlingmaier M Biller BMK Boguszewski MCS. Diagnosis, Genetics, and Therapy of Short Stature in Children: A Growth Hormone Research Society International Perspective. Horm Res Paediatr (2019) 92:1–14. doi: 10.1159/000502231 PMC6979443 31514194

[7] Grimberg A DiVall SA Polychronakos C Allen DB Cohen LE Quintos JB. Drug and Therapeutics Committee and Ethics Committee of the Pediatric Endocrine Society. Guidelines for Growth Hormone and Insulin-Like Growth Factor-I Treatment in Children and Adolescents: Growth Hormone Deficiency, Idiopathic Short Stature, and Primary Insulin-Like Growth Factor-I Deficiency. Horm Res Paediatr (2016) 86:361–97. doi: 10.1159/000452150 27884013

[8] Badaru A Wilson D. Alternatives to Growth Hormone Stimulation Testing in Children. Trends Endocrinol Metab (2004) 15:252–8. doi: 10.1016/j.tem.2004.06.004 15358277

[9] Tenenbaum-Rakover Y Hujeirat Y Admoni O Khayat M Allon-Shalev S Hess O. Can Auxology, IGF-I and IGFBP-3 Measurements Followed by MRI and Genetic Tests Replace GH Stimulation Tests in the Diagnosis of GH Deficiency in Children? J Pediatr Endocrinol Metab (2010) 23(4):387–94. doi: 10.1515/jpem.2010.060 20583544

[10] Rosenfeld RG Albertsson-Wikland K Cassorla F Frasier SD Hasegawa Y Hintz RL. Diagnostic Controversy: The Diagnosis of Childhood Growth Hormone Deficiency Revisited. J Clin Endocrinol Metab (1995) 80:1532–40. doi: 10.1210/jcem.80.5.7538145 7538145

[11] Rosenfeld RG. Is Growth Hormone Deficiency a Viable Diagnosis? J Clin Endocrinol Metab (1997) 82:349–51. doi: 10.1210/jcem.82.2.3841. D. M. 9024216

[12] Møller N Jørgensen JOL. Effects of Growth Hormone on Glucose, Lipid, and Protein Metabolism in Human Subjects. Endocrine Rev (2009) 30:152–77. doi: 10.1210/er.2008-0027 19240267

[13] Mamilly L Pyle-Eilola AL Chaudhari M Henry RK. The Utility of a Random Growth Hormone Level in Determining Neonatal Growth Hormone Sufficiency. Clin Endocrinol (2021) 94:392–8. doi: 10.1111/cen.14364 33140844

[14] Dehkhoda F Lee CMM Medina J Brooks AJ. The Growth Hormone Receptor: Mechanism of Receptor Activation, Cell Signaling, and Physiological Aspects. Front Endocrinol (2018) 9:35. doi: 10.3389/fendo.2018.00035 PMC5816795 29487568

[15] Purandare A Co Ng L Godil M Ahnn SH Wilson TA. Effect of Hypothyroidism and Its Treatment on the IGF System in Infants and Children. J Pediatr Endocrinol Metab (2003) 16(1):35–42. doi: 10.1515/JPEM.2003.16.1.35 12585338

[16] Jansson U Kristiansson B Magnusson P Larsson L Albertsson-Wikland K Bjarnason R. The Decrease of IGF-I, IGF-Binding Protein-3 and Bone Alkaline Phosphatase Isoforms During Gluten Challenge Correlates With Small Intestinal Inflammation in Children With Coeliac Disease. Eur J Endocrinol (2001) 144(2):417–23. doi: 10.1530/eje.0.1440417 11275953

[17] Støving RK Hangaard J Hagen C Flyvbjerg A. Low Levels of the 150-kD Insulin-Like Growth Factor Binding Protein 3 Ternary Complex in Patients With Anorexia Nervosa: Effect of Partial Weight Recovery. Horm Res Paediatr (2003) 60:43–8. doi: 10.1159/000070826 12792153

[18] Wit JM Bidlingmaier M de Bruin C Oostdijk W. A Proposal for the Interpretation of Serum IGF-I Concentration as Part of Laboratory Screening in Children With Growth Failure. Jcrpe (2020) 12:130–9. doi: 10.4274/jcrpe.galenos.2019.2019.0176 PMC7291410 31842524

[19] Hawkes CP Grimberg A. Insulin-Like Growth Factor-I Is a Marker for the Nutritional State. Pediatr Endocrinol Rev (2015) 13:499–511. 26841638 PMC5576178

[20] Binder G Weidenkeller M Blumenstock G Langkamp M Weber K Franz AR. Rational Approach to the Diagnosis of Severe Growth Hormone Deficiency in the Newborn. J Clin Endocrinol Metab (2010) 95:2219–26. doi: 10.1210/jc.2009-2692 20332247

[21] Bidlingmaier M Friedrich N Emeny RT Spranger J Wolthers OD Roswall J. Reference Intervals for Insulin-Like Growth Factor-1 (IGF-I) From Birth to Senescence: Results From a Multicenter Study Using a New Automated Chemiluminescence IGF-I Immunoassay Conforming to Recent International Recommendations. J Clin Endocrinol Metab (2014) 99:1712–21. doi: 10.1210/jc.2013-3059 24606072

[22] Growth Hormone Research Society Consensus Guidelines for the Diagnosis and Treatment of Growth Hormone (GH) Deficiency in Childhood and Adolescence: Summary Statement of the GH Research Society. J Clin Endocrinol Metab (2000) 85:3990–3. doi: 10.1210/jcem.85.11.6984. 11095419

[23] Cohen P Rogol AD Deal CL Saenger P Reiter EO Ross JL. Consensus Statement on the Diagnosis and Treatment of Children With Idiopathic Short Stature: A Summary of the Growth Hormone Research Society, the Lawson Wilkins Pediatric Endocrine Society, and the European Society for Paediatric Endocrinology Workshop. J Clin Endocrinol Metab (2008) 93:4210–7. doi: 10.1210/jc.2008-0509 18782877

[24] Binder G Reinehr T Ibáñez L Thiele S Linglart A Woelfle J. GHD Diagnostics in Europe and the US: An Audit of National Guidelines and Practice. Horm Res Paediatr (2019) 92:150–6. doi: 10.1159/000503783 31707392

[25] Juul A Bernasconi S Clayton PE Kiess W DeMuinck-Keizer Schrama S. European Audit of Current Practice in Diagnosis and Treatment of Childhood Growth Hormone Deficiency. Horm Res Paediatr (2002) 58:233–41. doi: 10.1159/000066265 12401943

[26] Clément F Grinspon RP Yankelevich D Martín Benítez S de la Ossa Salgado MC Ropelato MG. Development and Validation of a Prediction Rule for Growth Hormone Deficiency Without Need for Pharmacological Stimulation Tests in Children With Risk Factors. Front Endocrinol (2021) 11:624684. doi: 10.3389/fendo.2020.624684 PMC7887303 33613456

[27] Kim JH Chae HW Chin SO Ku CR Park KH Lim DJ. Diagnosis and Treatment of Growth Hormone Deficiency: A Position Statement From Korean Endocrine Society and Korean Society of Pediatric Endocrinology. Endocrinol Metab (2020) 35:272–87. doi: 10.3803/EnM.2020.35.2.272 PMC7386113 32615711

[28] Felício JS Janaú LC Moraes MA Zahalan NA de Souza Resende F de Lemos MN. Diagnosis of Idiopathic GHD in Children Based on Response to rhGH Treatment: The Importance of GH Provocative Tests and IGF-1. Front Endocrinol (2019) 10:638. doi: 10.3389/fendo.2019.00638 PMC6763693 31616374

[29] Murray PG Dattani MT Clayton PE. Controversies in the Diagnosis and Management of Growth Hormone Deficiency in Childhood and Adolescence. Arch Dis Childhood (2016) 101:96–100. doi: 10.1136/archdischild-2014-307228 26153506

[30] Clemmons DR. Consensus Statement on the Standardization and Evaluation of Growth Hormone and Insulin-Like Growth Factor Assays. Clin Chem (2011) 57:555–9. doi: 10.1373/clinchem.2010.150631 21285256

[31] Binder G Huller E Blumenstock G Schweizer R. Auxology-Based Cut-Off Values for Biochemical Testing of GH Secretion in Childhood. Growth Hormone IGF Res (2011) 21:212–8. doi: 10.1016/j.ghir.2011.05.007 21665508

[32] Guzzetti C Ibba A Pilia S Beltrami N Di Iorgi N Rollo A. Cut-Off Limits of the Peak GH Response to Stimulation Tests for the Diagnosis of GH Deficiency in Children and Adolescents: Study in Patients With Organic GHD. Eur J Endocrinol (2016) 175:41–7. doi: 10.1530/EJE-16-0105 27147639

[33] Sobrier M-L Maghnie M Vié-Luton M-P Secco A di Iorgi N Lorini R. Novel HESX1 Mutations Associated With a Life-Threatening Neonatal Phenotype, Pituitary Aplasia, But Normally Located Posterior Pituitary and No Optic Nerve Abnormalities. J Clin Endocrinol Metab (2006) 91:4528–36. doi: 10.1210/jc.2006-0426 16940453

[34] Pollock NI Cohen LE. Growth Hormone Deficiency and Treatment in Childhood Cancer Survivors. Front Endocrinol (2021) 12:745932. doi: 10.3389/fendo.2021.745932 PMC8569790 34745010

[35] Sklar CA Antal Z Chemaitilly W Cohen LE Follin C Meacham LR. Hypothalamic–Pituitary and Growth Disorders in Survivors of Childhood Cancer: An Endocrine Society* Clinical Practice Guideline. J Clin Endocrinol Metab (2018) 103:2761–84. doi: 10.1210/jc.2018-01175 29982476

[36] Dassa Y Crosnier H Chevignard M Viaud M Personnier C Flechtner I. Pituitary Deficiency and Precocious Puberty After Childhood Severe Traumatic Brain Injury: A Long-Term Follow-Up Prospective Study. Eur J Endocrinol (2019) 180:281–90. doi: 10.1530/EJE-19-0034 30884465

[37] Laron Z Mannheimer S Pertzelan A Nitzan M. Serum Growth Hormone Concentration in Full Term Infants. Isr J Med Sci (1966) 2:770–3. 5956694

[38] Cornblath M Parker ML Reisner SH Forbes AE Daughaday WH. Secretion and Metabolism of Growth Hormone in Premature and Full-Term Infants1. J Clin Endocrinol Metab (1965) 25:209–18. doi: 10.1210/jcem-25-2-209 14264242

[39] Binder G Weber K Rieflin N Steinruck L Blumenstock G Janzen N. Diagnosis of Severe Growth Hormone Deficiency in the Newborn. Clin Endocrinol (2020) 93(3):305–11. doi: 10.1111/cen.14264 32521075

[40] Kelly A Tang R Becker S Stanley CA. Poor Specificity of Low Growth Hormone and Cortisol Levels During Fasting Hypoglycemia for the Diagnoses of Growth Hormone Deficiency and Adrenal Insufficiency. PEDIATRICS (2008) 122:e522–8. doi: 10.1542/peds.2008-0806 18694902

[41] Dominguez-Menéndez G Cifuentes L González C Lagos M Quiroga T Rumié H. Hormona De Crecimiento En Sangre De Papel Filtro Para El Diagnóstico De Deficiencia De Hormona De Crecimiento. Rev Chil Pediatr (2019) 90:145. doi: 10.32641/rchped.v90i2.674 31095230

[42] Cianfarani S Tondinelli T Spadoni GL Scire G Boemi S Boscherini B. Height Velocity and IGF-I Assessment in the Diagnosis of Childhood Onset GH Insufficiency: Do We Still Need a Second GH Stimulation Test? Clin Endocrinol (2002) 57:161–7. doi: 10.1046/j.1365-2265.2002.01591.x 12153594

[43] Shen Y Zhang J Zhao Y Yan Y Liu Y Cai J. Diagnostic Value of Serum IGF-1 and IGFBP-3 in Growth Hormone Deficiency: A Systematic Review With Meta-Analysis. Eur J Pediatr (2015) 174:419–27. doi: 10.1007/s00431-014-2406-3 25213432

[44] Study Group on Physiopathology of growth processes Council of ISPED Federico G Street ME Maghnie M Caruso-Nicoletti M. Assessment of Serum IGF-I Concentrations in the Diagnosis of Isolated Childhood-Onset GH Deficiency: A Proposal of the Italian Society for Pediatric Endocrinology and Diabetes (SIEDP/ISPED). J Endocrinological Invest (2006) 29:732–7. doi: 10.1007/BF03344184 17033263

[45] Wang Y Zhang H Cao M Kong L Ge X. Analysis of the Value and Correlation of IGF−1 With GH and IGFBP−3 in the Diagnosis of Dwarfism. Exp Ther Med (2019) 17(5):3689–93. doi: 10.3892/etm.2019.7393 PMC6447816 30988753

[46] Boquete HR Sobrado PGV Fideleff HL Sequera AM Giaccio AV Suárez MG. Evaluation of Diagnostic Accuracy of Insulin-Like Growth Factor (IGF)-I and IGF-Binding Protein-3 in Growth Hormone-Deficient Children and Adults Using ROC Plot Analysis. J Clin Endocrinol Metab (2003) 88:4702–8. doi: 10.1210/jc.2003-030412 14557444

[47] Bussières L Souberbielle J-C Pinto G Adan L Noel M Brauner R. The Use of Insulin-Like Growth Factor 1 Reference Values for the Diagnosis of Growth Hormone Deficiency in Prepubertal Children: IGF-1 Reference Values. Clin Endocrinol (2000) 52:735–9. doi: 10.1046/j.1365-2265.2000.00999.x 10848878

[48] Cianfarani S Liguori A Germani D. IGF-I and IGFBP-3 Assessment in the Management of Childhood Onset Growth Hormone Deficiency. In: Cianfarani S Clemmons DR Savage MO, editors. Endocrine Development. Basel: KARGER (2005). p. 66–75. doi: 10.1159/000085757 15879689

[49] Bogazzi F Manetti L Lombardi M Giovannetti C Raffaelli V Urbani C. Impact of Different Cut-Off Limits of Peak GH After GHRH-Arginine Stimulatory Test, Single IGF1 Measurement, or Their Combination in Identifying Adult Patients With GH Deficiency. Eur J Endocrinol (2011) 164:685–93. doi: 10.1530/EJE-10-1068 21307143

[50] Inoue-Lima TH Vasques GA Scalco RC Nakaguma M Mendonca BB Arnhold IJP. IGF-1 Assessed by Pubertal Status has the Best Positive Predictive Power for GH Deficiency Diagnosis in Peripubertal Children. J Pediatr Endocrinol Metab (2019) 32:173–9. doi: 10.1515/jpem-2018-0435 30676998

[51] Federico G Cianfarani S. Usefulness of Serum Insulin-Like Growth Factor I Assessment in the Diagnosis of Childhood-Onset Growth Hormone Deficiency. Hormone Res Paediatrics (2010) 74:145–8. doi: 10.1159/000314895 20551620

[52] Alawneh H Khaledi O Maita J. Insulin Like Growth Factor 1 as an Indicator of Growth Hormone Deficiency. JRMS (2015) 22:13–7. doi: 10.12816/0011355

[53] Ibba A Corrias F Guzzetti C Casula L Salerno M di Iorgi N. IGF1 for the Diagnosis of Growth Hormone Deficiency in Children and Adolescents: A Reappraisal. Endocrine Connections (2020) 9:1095–102. doi: 10.1530/EC-20-0347 PMC7774770 33112822

[54] Cianfarani S Liguori A Boemi S Maghnie M Iughetti L Wasniewska M. Inaccuracy of Insulin-Like Growth Factor (IGF) Binding Protein (IGFBP)-3 Assessment in the Diagnosis of Growth Hormone (GH) Deficiency From Childhood to Young Adulthood: Association to Low GH Dependency of IGF-II and Presence of Circulating IGFBP-3 18-Kilodalton Fragment. J Clin Endocrinol Metab (2005) 90:6028–34. doi: 10.1210/jc.2005-0721 16091488

[55] Phillip M Chalew SA Kowarski AA Stene MA. Plasma IGFBP-3 and Its Relationship With Quantitative Growth Hormone Secretion in Short Children. Clin Endocrinol (1993) 39:427–32. doi: 10.1111/j.1365-2265.1993.tb02389.x 7507010

[56] Hage C Gan H-W Ibba A Patti G Dattani M Loche S. Advances in Differential Diagnosis and Management of Growth Hormone Deficiency in Children. Nat Rev Endocrinol (2021) 17:608–24. doi: 10.1038/s41574-021-00539-5 34417587

[57] Iorgi ND Allegri AEM Napoli F Bertelli E Olivieri I Rossi A. The Use of Neuroimaging for Assessing Disorders of Pituitary Development: Assessing Disorders of Pituitary Development. Clin Endocrinol (2012) 76:161–76. doi: 10.1111/j.1365-2265.2011.04238.x 21955099

[58] Kalina MA Kalina-Faska B Gruszczyńska K Baron J Małecka-Tendera E. Usefulness of Magnetic Resonance Findings of the Hypothalamic-Pituitary Region in the Management of Short Children With Growth Hormone Deficiency: Evidence From a Longitudinal Study. Childs Nerv Syst (2012) 28:121–7. doi: 10.1007/s00381-011-1594-7 PMC3252499 21935593

[59] Wit JM Oostdijk W Losekoot M van Duyvenvoorde HA Ruivenkamp CAL Kant SG. Mechanisms in Endocrinology: Novel Genetic Causes of Short Stature. Eur J Endocrinol (2016) 174:R145–73. doi: 10.1530/EJE-15-0937 26578640

